# Monitoring the impact of cannabis use

**DOI:** 10.47626/2237-6089-2021-0462

**Published:** 2022-06-27

**Authors:** Bernard Le Foll

**Affiliations:** 1 CAMH Toronto ON Canada Centre for Addiction and Mental Health (CAMH), Toronto, ON, Canada. Departments of Psychiatry, Family and Community Medicine, and Pharmacology and Toxicology, University of Toronto, Toronto, ON, Canada.; 2 Departments of Psychiatry, Family and Community Medicine, and Pharmacology and Toxicology University of Toronto Toronto ON Canada; 3 Institute of Medical Science University of Toronto Toronto ON Canada Institute of Medical Science, University of Toronto, Toronto, ON, Canada.

Cannabis is widely used in the world.^1^ For example, it has been estimated that 183 million people used cannabis in 2014. Multiple countries have authorized the use of cannabis for medical reasons and some countries have legalized non-medical cannabis use. Users can now get easy access to legal cannabis for recreational use in Canada, Uruguay and some states in the United States of America and the list of countries that are thinking of legalizing cannabis is growing. It is clear that legalizing cannabis can provide some benefits (for example, having a regulated supply with quality controls, preventing sales to youth, reducing the money going to drug cartels and providing those revenues to the public coffers, and reducing the court workload). It also can produce some risks (for example, increased overall use in the population that may be associated with increased harm, notably through cannabis use disorder, increased traffic collisions, mental health impact, and increased intoxications). In this context, this special supplement of the journal *Trends in Psychiatry and Psychotherapy* on Cannabis organized by Drs. Thiago Fidalgo and Lisia von Diemen is very timely.

In this special issue, Barata et al. discuss the different models for legalization and the international policies that could be implemented to prevent harm.^2^ In another article, Ransing et al. discuss and compare the situation related to cannabis use in sixteen countries.^3^ It is clear that if we want to promote public health, the experience arising from countries that have legalized cannabis is critical, although we are still in the early years following the legalization of cannabis in those countries and it is too early to conclude on the impact of cannabis legalization. However, it will be important to monitor the situation carefully in the next few years to be able to determine this impact with greater confidence. There will be interesting comparisons to make between countries/jurisdictions that have legalized cannabis. For example, the model of legalization in Colorado has put less emphasis on public health as compared the model of legalization used in Canada. Early analysis suggests a higher increase of cannabis use in young adults after legalization in Colorado, as compared to Canada.^4,5^ Such differences would support the importance of prioritizing a public health lens while implementing legalization of cannabis use.

Such legalization process should also take into consideration the various factors that underlie cannabis use. Epidemiological studies have indicated that most of the factors that are associated with the risk of developing cannabis use disorder are similar to those for other drugs of abuse. For example, known factors for developing cannabis use disorders are: initiation of use by age 15, low socioeconomic status, personal or peer use of other drugs and tobacco, regular cannabis use, anti-social behavior, using cannabis as a coping mechanism, lack of knowledge of risk or norm misperceptions, concurrent mental health disorders, poor parental monitoring and supervision, low family bonding, high family conflict, and lack of school engagement.^6^ In this special issue, Dias et al. also highlight the importance of the mediating role of friends’ use in cannabis abuse.^7^

The negative impact of cannabis use in populations is primarily driven by (in order of importance): 1) cannabis use disorder; 2) impact on driving; and 3) intoxication and mental health impact.^8^ The risk for high potency cannabis in particular to develop cannabis-induced psychosis has attracted lots of attention. Similarly, reducing the possible impact of cannabis use on driving and related traffic collision has been a big priority in Canada following legalization. Not surprisingly, this area is currently an active area of research.^9^ But clearly, cannabis use disorder is likely the complication that will require the most investment in the near future due to its importance.

It is estimated that around 9 percent of those who initiate cannabis use will develop dependence later on in life.^1^ It should be noted that an even larger proportion would develop cannabis use disorder at some point of their life. Cannabis use is defined by the American Psychiatric Association as a pattern of cannabis use that causes clinically significant psychiatric distress and social impairment, as well as multiple adverse consequences and repeated unsuccessful attempts to quit.^10^ It is likely that increase of cannabis use in the population may be subsequently followed by an increased incidence of cannabis use disorder. With the increasing use of cannabis, there has already been an increasing demand for specific treatments for cannabis use disorder in North America. At the present time, very few centers have developed or implemented treatments that are specific for cannabis users. However, it is important to note that significant research has been done in this area of care.^11,12^ These research studies indicate that psychosocial interventions are effective for management of cannabis use disorder.^12^ The most effective treatments available are based on cognitive-behavioral therapy (CBT) and motivation enhancement therapy (MET). These interventions can be delivered in person or in groups. It should be noted that contingency management is also effective. However, unfortunately the latter is rarely used outside of research settings. Combining these various interventions seems beneficial.^12,13-15^ A series of trials have investigated the utility of pharmacotherapies for management of cannabis use disorders. It is important to note that at the present time there are no approved pharmacotherapies for this indication.^11^ However, clinicians have sometimes used pharmacotherapies for management of various phases of treatment. Notably, there are patients that can benefit from the use of pharmacotherapies for management of cannabis withdrawal symptoms.^16^ For long-term management of cannabis use disorder, medications such as antidepressants, anxiolytics, mood stabilizers, and antiepileptic drugs have no utility. The most promising agents appear to be medications that stimulate the endocannabinoid system, either directly as cannabinoid CB1 agonists (for example, nabiximols^17,18^ or nabilone), or indirectly by blocking the Fatty Acid Amide Hydrolase enzyme that degrades anandamide.^19^ However, these approaches are still experimental and not fully validated. It is likely that in the near future, pharmacotherapies will be develop and validated for this indication.

To conclude, it is an interesting time for cannabis research and treatment. The next few years, will likely inform us of the real impact of cannabis legalization and that will have some importance to guide future policy decisions. It is also clear that treatment providers and health care systems should already plan for an increased demand for specialized treatment for cannabis use disorder and it would be beneficial to start implementing specialized evidence-based program to adequately respond to this growing clinical demand.
